# Metabolic characterization of the new benzimidazole synthetic opioids - nitazenes

**DOI:** 10.3389/fphar.2024.1434573

**Published:** 2024-07-18

**Authors:** Gajanan R. Jadhav, Pius S. Fasinu

**Affiliations:** Department of Pharmacology and Toxicology, Heersink School of Medicine, The University of Alabama at Birmingham, Birmingham, AL, United States

**Keywords:** benzimidazole, butonitazene, cytochrome P450 enzymes, drug metabolism, isotonitazene, new synthetic opioids, nitazenes, protonitazene

## Abstract

The recent re-emergence and the increasing popularity of nitazenes, a group of new synthetic opioids (NSO) that belong to the benzimidazole chemical class, has raised public health concerns. As a class of potential opioid analgesic agents whose development was discontinued in the 1960s due to their high potential for abuse, very little is known about their metabolism and physiologic disposition. In the current study, three nitazenes–butonitazene, isotonitazene and protonitaze were incubated in human liver microsomes (HLM), human S9 (HS9) fractions and recombinant cytochrome P450 enzymes. All three nitazenes were rapidly metabolized in both HLM and HS9 with over 95% depletion within 60 min. In HLM, butonitazene, isotonitazene and protonitazene had *in vitro* intrinsic clearance (CLint) (µL/min/mg protein) values of 309, 221 and 216 respectively compared to 150 of verapamil, the positive control. In HS9, CLint values were 217, 139, and 150 for butonitazene, isotonitazene and protonitazene respectively compared to only 35 for testosterone, the control probe substrate. Putative metabolite identified from this study include products of hydroxylation, desethylation, dealkylation, desethylation followed by dealkylation, and desethylation followed by hydroxylation. The metabolic phenotyping showed CYP2D6, CYP2B6 and CYP2C8 and the major hepatic enzymes responsible for the metabolism of nitazenes. Within 30 min of incubation, CYP2D6 depleted butonitazene (99%), isotonitazene (72%) and butonitazene (100%) significantly. The rapid metabolism of nitazenes may be an important factor in accurate and timely detections and quantitation of the unchanged drugs in human matrices following intoxication or in forensic analysis. The involvement of multiple polymorphic CYPs in their metabolism may play important roles in the susceptibility to intoxication and/or addiction, depending on the activity of the metabolites.

## Introduction

The emergence of new psychoactive substances (NPS) over the past 2 decades has raised public health concerns globally (Hall and Miczek, 2019; Peacock et al., 2019; Rinaldi et al., 2020; Simão et al., 2022). According to the United Nations Office on Drugs and Crime, more than a thousand reports on NPS including synthetic opioids, psychostimulants, hallucinogens, and depressants were received between 2009 and 2021 (UNODCUnited Nations Office on Drugs and CrimeWorld Drug Report, 2022). Of these NPS, the fastest growing group in North America and Europe is the new synthetic opioids (NSO). Not only are NSOs potentially the most harmful because of their high potency, they have continued to grow in number in multiple states in contrast to the general decline in the number of other NPS (DEA. United States Drug Enforcement Administration, 2022). Along with prescription and illicitly procured opioids, NSOs are widely abused in the US, causing over 107,000 deaths in 2021, with an estimated economic cost of over $1.02 trillion annually (CDC, 2021; Florence et al., 2021). While the pharmacologic effects, metabolic profiles and dose-response of traditional opioids are well understood, little or no information is available on recently identified NSOs. This knowledge gap presents a significant challenge in early detection and treatment of intoxication.

NSOs are generally categorized into ‘fentalogues’ (fentanyl and its analogues) and the non-fentanyls. The most poorly understood and poorly studied are non-fentanyl NSOs (nf-NSO). The nf-NSOs are chemically classified into nitazenes (also called benzimidazoles), diphenylethylpiperazines, cyclohexylbenzamides, cinnaylpiperazines, and benzimidazolones (Karila et al., 2019; Zawilska et al., 2023). First synthesized as designer opioids in the 1950s by CIBA Aktiengesellschaf, a Swiss pharmaceutical company in search of alternative analgesic agents, nitazenes were quickly discovered to have high abuse potential. The development was halted, and no nitazene was approved for human use. In the 1960s, only etonitazene and clonitazene were scheduled in the United States under the Controlled Substances Act (United Nations, 1961). Since 2019, several nitazenes have been detected in toxicological samples and contaminated illicit drugs. Based on the reports by the US Drug Enforcement Agency (DEA) and the Center for Forensic Science Research and Education (CFSRE), nitazenes, unlike many emergent drugs of abuse, have persisted in toxicological samples and forensic laboratory analyses since the 2019 emergence (CCENDU, 2022; DEA. United States Drug Enforcement Administration, 2022; Papsun, et al., 2022; CFSRE, 2024). Having recognized these substances as threats to public health, the DEA initially placed isotonitazene in the schedule I category in 2020 and later added seven more nitazenes including butonitazene, etodesnitazene (etazene), flunitazene, metodesnitazene, metonitazene, etonitazepyne (pyrrolidino etonitazene) and protonitazene, emphasizing their strong potential for abuse and exclusion from any human use (US Federal Register, 2021). Since the discontinuation of their development in the 1950s, few scientific studies have been conducted on nitazenes until their 2019 re-emergence. Thus, information available on this class of drugs is largely from the early discovery studies.

Nitazenes are some of the most potent opioids. The discovery studies comparing their potency to morphine, the standard reference opioid, showed potency up to 500 times that of morphine (Ujvary et al., 2021; Vandeputte et al., 2020; Vandeputte et al., 2021; Vandeputte et al., 2022; Vandeputte et al., 2023; Stucke et al., 2023). Despite the huge public health concern that nitazenes constitutes, there is a wide knowledge gap in their metabolism and toxicokinetics. As benzimidazole derivatives, nitazenes are chemically distinct from the three major opioid chemical classes: phenanthrenes (like morphine, codeine), phenylpiperidines (fentalogues) and diphenylheptanes (methadone). Based on different substitutions on the benzimidazole ring (Table 1), over 20 nitazenes have been identified globally. The phenanthrenes are substrates of CYP2D6 while the phenylpiperidines and diphenylheptanes are primarily metabolized by CYP3A4. The knowledge of the metabolic pathways of other opioids is therefore not sufficient to predict how nitazenes are metabolized. CYP2D6 is highly polymorphic and its genotypic expressions in individuals largely affect their response to pain management. Additionally, many of the drugs in the phenanthrene class, including codeine and hydrocodone, are prodrugs; their activity relies on CYP2D6-dependent bioactivation. The metabolic characterization of nitazenes, in a similar way, can enhance the understanding of the roles of the different enzymes their inactivation/bioactivation and intoxication. The evaluation of the metabolic pathways of nitazenes is important in estimating the genetic influences on intoxication and addiction susceptibilities. Thirdly, our understanding of the metabolic pathways and metabolic identities of nitazenes can improve the accuracy of laboratory determinations in forensic and pathological analyses.

**TABLE 1:** Molecular structures of benzimidazole opioids (nitazenes).

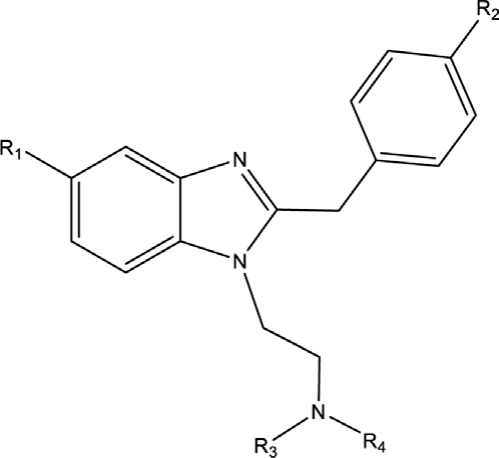				
Nitazene	R1	R2	R3	R4
4-Hydroxy nitazenes	NO_2_	OH	CH_2_CH_3_	CH_2_CH_3_
5-Amino Isotonitazene	NH2	OCH(CH_3_)_2_	CH_2_CH_3_	CH_2_CH_3_
Butonitazene	NO_2_	OCH_2_CH_2_CH_2_CH_3_	CH_2_CH_3_	CH_2_CH_3_
Clonitazene	NO_2_	Cl	CH_2_CH_3_	CH_2_CH_3_
N-Desethylisonitazene	NO_2_	OCH(CH_3_)_2_	-	CH_2_CH_3_
N-Desetyletonitazene	NO_2_	OCH_2_CH_3_	CH_2_CH_3_	-
Etodesnitazene	H	OCH_2_CH_3_	CH_2_CH_3_	CH_2_CH_3_
Etomethazene	H	OCH_2_CH_3_	CH_2_CH_3_	CH_2_CH_3_
Etoetonitazene	NO_2_	OCH2CH2OCH2CH3	CH_2_CH_3_	CH_2_CH_3_
Etonitazene	NO_2_	OCH_2_CH_3_	CH_2_CH_3_	CH_2_CH_3_
Etonitazepipne	NO_2_	OCH_2_CH_3_	-CH_2_CH_2_CH_2_CH_2_-	
Etonitazepyne	NO_2_	OCH_2_CH_3_	-CH_2_CH_2_CH_2_CH_2_-	
Flunitazene	NO_2_	F	CH_2_CH_3_	CH_2_CH_3_
Isotodesnitazene	H	OCH(CH_3_)_2_	CH_2_CH_3_	CH_2_CH_3_
Isotonitazene	NO_2_	OCH(CH_3_)_2_	CH_2_CH_3_	CH_2_CH_3_
Methylthionitazene	NO_2_	SCH_3_	CH_2_CH_3_	CH_2_CH_3_
Metodesnitazene	H	OCH_3_	CH_2_CH_3_	CH_2_CH_3_
Metonitazene or α-Methylmetonitazene	NO_2_	OCH_3_	CH_2_CH_3_	CH_2_CH_3_
N-piperidino etonitazene	NO_2_	OCH2CH3	--CH_2_CH_2_CH_2_CH_2_-	
Propylnitazene	NO_2_	CH_2_CH_2_CH_3_	CH_2_CH_3_	CH_2_CH_3_
Protonitazene	NO_2_	OCH_2_CH_2_CH_3_	CH_2_CH_3_	CH_2_CH_3_

The aim of the current study was to evaluate three nitazenes - butonitazene, isotonitazene and protonitazene for *in vitro* metabolic stability utilizing human liver microsomes and liver S9 fractions. The study also assessed metabolic reaction phenotyping using recombinant cytochrome P450 enzymes. It was designed to provide the initial metabolism data towards the understanding of the pharmacokinetic and toxicokinetic profiles of nitazenes.

## Materials and methods

Butonitazene, isotonitazene and protonitazene (preparations of 1 mg/mL in methanol), and diltiazem hydrochloride were purchased from Cayman Chemical Company (Ann Arbor, MI, USA), Human liver microsomes (HLM) (mixed gender, pool of 50), human liver S9 (mixed gender, pool of 50), recombinant CYP1A2, CYP2A6, CYP2B6, CYP2C8, CYP2C9, CYP2C19, CYP2D6, CYP2E1 and CYP3A4 were procured from BioIVT (Westbury, NY, USA). Sodium phosphate (monobasic and dibasic), Nicotinamide adenine dinucleotide phosphate (NADP) testosterone, and verapamil were purchased from Sigma-Aldrich (St. Louis, MO, USA). Acetonitrile and methanol (HPLC-grade) were purchased from Fisher Scientific (Fair Lawn, NJ, USA).

### Microsomal incubation

Thawed HLMs were diluted with sodium phosphate buffer (50 mM; pH 7.4) and incubated in 96-well plates. Prepared solutions (100 µM) of nitazenes and positive controls were added to the microsomal mixture. Reaction mixture was preincubated for 10 min at 37 °C and 60 RPM. A start solution made of 10 mM NADPH was added to initiate metabolic reaction. Control incubations were performed without the cofactor NADPH solution. The final concentration of methanol was less than 0.5% in all the incubations. All incubations were performed in duplicates. The incubation mixture was sampled at 0, 5, 10, 15, 30 and 60 min and immediately added to ice-cold methanol to precipitate the microsomal proteins and stop metabolic reactions. Pending analysis, reaction samples were stored at −80 °C deep freezer.

For analysis, samples were retrieved, mixed well, and centrifuged at 10000 RPM, at 4°C for 20 min. Aliquots of the clear supernatants (80 µL) were transferred to the autosampler vials for LC-MS/MS analysis. Quantitative and qualitative analysis were performed using the LC-MS/MS API-4500 triple quadrupole, through the application of the methods described below.

### Incubation with human liver S9 fractions

The incubation mixture was prepared by diluting the human liver S9 fractions with sodium phosphate buffer (pH 7.4). The final incubation volume of 500 µL was made of 385 µL mixed buffer, 12.5 µL of human liver S9, 50 µL of NADPH, 50 µL of UDPGA and 2.5 µL substrate. Before the addition of the substrate, the reaction mixture was preincubated for 10 min at 37 °C and 60 RPM. The final concentration of the substrate and positive control was 100 µM. All incubations were performed in duplicates. Samples (50 µL) were collected at 0, 15 and 60 min in 100 µL of methanol and stored at −80 °C freezer until analysis. The samples were prepared as described in the microsomal study and analysis was performed by LC-MS/MS API-4500 quadrupole using the analytical methodology described below.

### Reaction phenotyping in recombinant cytochrome P450 enzyme proteins

Similar to microsomal incubations, the metabolic reactions were set up in clear 96-well plates containing the thawed purified CYP supersomes diluted in sodium phosphate buffers (50 mM; pH 7.4). Performed in triplicates, each well contained 845 µL of the buffer, 50 µL of the purified 1 nmol CYPs, and 100 µL of NADPH solution. Reaction mixture was preincubated for 10 min at 37°C and 60 RPM. Reactions were initiated by the addition of 5 µL of substate or positive control solution (100 µM). Samples (50 µL) were collected at 0, 3, 6, 9, 12, 15, 20 and 30 min into ice-cold methanol (100 µL) to terminate metabolic reactions and were stored at −80°C till analysis. Negative control incubations were performed without cofactor NADPH solutions. For analysis, samples were retrieved, prepared, and analyzed as described in previous section using the LC-MS/MS API-4500 quadrupole method illustrated below.

### Analysis and metabolite identification

Liquid chromatography–mass spectrometry method was developed for the simultaneous analysis of butonitazene, isotonitazene and protonitazene. Total separation and elution of analytes were achieved within 7 min retention time using the Phenomenex, Synergi, 50 * 2.0 mm, 2.5 µ, Polar RP, 100 A° column on an ABSciex API 4500 mass spectrometer and Schimadzu liquid chromatography to which a conditioned auto-sampler (at 5°C) was attached. Initially, nitazenes were infused separately into the mass spectrometer in a solution containing 80% methanol (v/v) to obtain the molecular ion (Q1) for each analyte. Product ion (MS2) fragmentation and multiple reaction monitoring (MRM) for selected product ions were performed in 80% methanol (v/v) to optimize different voltages. The optimized potential values are presented in Table 2. LC conditions were optimized for sensitivity, resolution, and peak shape. Analytes were eluted in gradient mode with mobile phases A (methanol with 0.1% formic acid) and B (5 mM ammonium formate with 0.1% formic acid). Applying a flow rate of 0.25 mL/mL, the controller was started at 0.01 min at 40% pump B concentration, maintained for 1 min, decreased to 30% for another minute, increased to 60% till 5.5 min, and then decreased to 40% in next 1 min. The analyte injection volume was 2 μL, using the Phenomenex, Synergi, 50 * 2.0 mm, 2.5 µ, Polar RP, 100 A° column, with the column oven temperature set at 25°C and for a total run time of 7.5 min.

**TABLE 2:** Mass spectrometry settings for the analysis of nitazenes and their metabolites.

	Butonitazene	Isotonitazene	Protonitazene
Q1/MS2	425.0/100.2, 425.0/117.0	411.1/100.2	411.5/100.2, 411.5/112.7
Declustering potential (DP)	100	100	107
Collision Energy (CE)	35, 25	25	36, 25
Entrance Potential (EP)	10	10	10
Collion cell exit potential (CXP)	7	10	8, 23
Collision Associated Dissociation	5		
Gas 1 (Gs 1)	30		
Gas 2 (Gs 2)	60		
Curtain Gas (CUR)	25		
Ion Spray voltage (IS)	5,500		
Temperature (C°)	500		

For metabolite profiling, samples were injected into LC-MS using 0.1% formic acid in water as an aqueous mobile phase and 0.1% formic acid in acetonitrile as an organic mobile phase with C18 stationary phase, Luna^®^, Omega, 1.6 µm, 100, 100 * 2.1 mm, Phenomenex (Torrance,CA) analytical column. The rinsing solution was 50% methanol. The ion spray voltage for positive and negative mode of ionisation were ± 5,000/4500 V and the declustering potential was ± 80. Gas 1 and Gas 2 values were set 40 psi along 25 psi as the curtain gas. The interface temperature was 400°C. Samples post-elution was subjected to scan range from m/z 50–1,000 Da and product ion scans over 50 msec intervals with collision energy (CE) start was 15 eV with stop CE of 35 eV. The spectra were centroided and de-isotoped by Analyst software (ABSciex, Toronto, Canada).

### Data analysis

Substrate depletion was determined by the quantitative differences between the metabolism samples and the corresponding zero-time samples. With the aid of Graphpad Prism software version 10.2 (University of Rochester, Rochester, NY, USA), metabolism rates were profiled against the incubation time from which metabolic kinetic parameters were determined. Putative metabolic pathways were proposed based on the metabolites identified.

## Results

### Method development

Protonation on the nitazenes were observed (molecular (Q1) ion [M + H] in the positive mode of ionization). Fragments, specifically, *N*-alkylated part (*m/z* 100) and benzimidazole core structure (*m/z* 117) were reproducible, indicating that these ions were captured as product ions during infusion in mass spectrometer. The optimized fit-for-purpose method showed sensitivity with the limit of detection (LOD) of 244 pg/mL and limit of quantitation (LOQ) of 977 pg/mL. A linear calibration curve was generated in the concentration range of ∼1–500 ng/mL with regressions (r) of 0.9975, 0.9888 and 0.9900 for butonitazene, isotonitazene and protonitazene respectively. With basic pKa and being lipophilic in nature, the nitazenes showed good elution and sensitivity with the reverse phase chromatography with C18 stationary segment and an acidic mobile phase.

### Metabolic stability in human liver microsomes and human liver S9

Butonitazene, isotonitazene and protonitazene were incubated at varying concentrations in both human liver microsomes and S9 fractions. Substrate depletion and metabolite generation were monitored using the LCMS method developed for this purpose. Data acquisition was performed using Analyst^®^ software 1.7 and the output of area ratio (analyte/internal standard) was used for the stability assessment. The % metabolism and % remaining were calculated at each time point relative to the area ratio of control (0-time samples) incubations (0% depletion).

The % remaining data at each time point was incorporated as an input to a one-phase exponential decay model to estimate the half-life of nitazenes using GraphPad prism software. From the calculated half-life and elimination rate constant, intrinsic clearance (µL/min per mg of protein) was estimated for each test items and positive controls (Eq. 1). The summary of the metabolic kinetics is provided in Table 3.

CLint (μL/min/mg of protein)=kx (reaction volume/t1/2) / mg of protein concentration

**TABLE 3:** *In vitro* metabolic kinetics of butonitazene, isotonitazene and protonitazene in human liver microsomes and S9 fractions.

Compound	Human liver microsomes			Human liver S9		
	Metabolism (%)	Half-life (min)	CLint (µL/min/mg protein)	Metabolism (%)	Half-life (min)	CLint (µL/min/mg protein)
Butonitazene	100	4	309	97	8	217
Isotonitazene	100	6	221	96	10	139
Protonitazene	100	6	216	97	9	150
Verapamil/Testosterone	96	9	150	47	40	35

**FIGURE 1:**
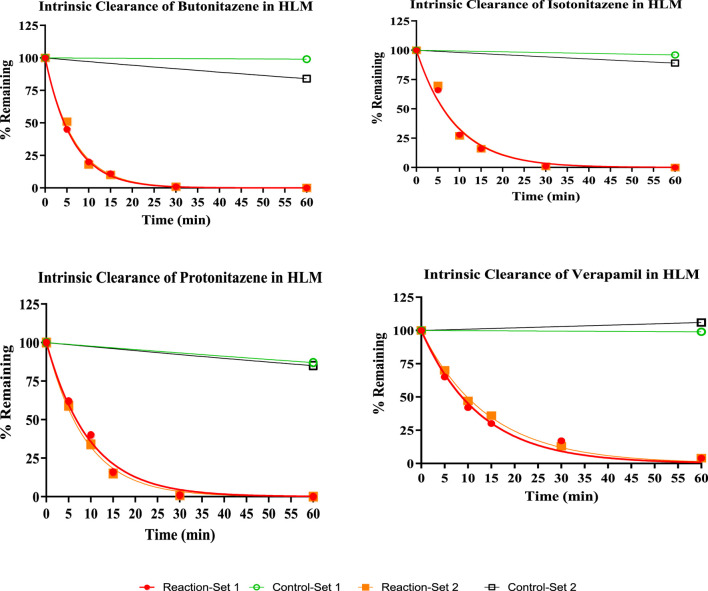
The depletion of nitazenes in human liver microsomes.

All three nitazenes were rapidly metabolized in both human liver microsomes and human liver S9 with almost compete depletion in 60 min. The rate of metabolism of the nitazenes was approximately double that of verapamil, the positive control in the liver microsomes (Figure 1). Rates were much higher in liver S9 fractions with butonitazene, for example, having a clearance value up to 6 times that of testosterone (Table 3). The major inference from these results is that nitazenes are substrates of liver enzymes, and it might be important to determine which liver enzymes are responsible or their metabolism.

### Metabolic reaction phenotyping

The depletion of the nitazenes was evaluated by comparing the concentration differences between the 0-time and 30-minute-time incubation in the recombinant CYPs. Butonitazene, isotonitazene and protonitazene demonstrated the most depletion in CYP2D6 (99%, 54% and 98% respectively) and CYP 2B6 (85%, 32% and 97% respectively). All three nitazenes also showed significant depletion in CYP2C8 (Table 4).

**TABLE 4:** The depletion of butonitazene, isotonitazene and protonitazene in recombinant cytochrome P450 enzymes.

CYP	Extent of metabolism after 30 min (%)			
	Control substrate^a^	Butonitazene	Isotonitazene	Protonitazene
CYP1A2	42	3	9	12
CYP2A6	81	20	32	25
CYP2B6	40	85	−5	97
CYP2C8	80	34	44	44
CYP2C9	95	17	−7	−6
CYP2C19	25	−9	0	16
CYP2D6	100	99	54	98
CYP2E1	80	26	42	42
CYP3A4	66	6	−12	5

^a^ Phenacetin (CYP1A2); Diclofenac (CYP2C9); S-Mephenytoin (CYP2C19); Dextromethorphan (CYP2D6); Testosterone (CYP3A4); Coumarin (CYP2A6); Bupropion (CYP2B6); Amodiaquine (CYP2C8); Chlorzoxazone (CYP2E1).

The three nitazenes showed strong substrate affinity for the CYPs, particularly CYP2D6, CYP2B6, CYP3A4 and CYP2C8 depleting faster than the control CYP-specific substrates utilized in the experiment. The metabolic kinetics were estimated from concentration-time profiles of the *in vitro* incubation with all three nitazenes demonstrating half-life values in single-digit minutes in CYP2D6. More detailed metabolic kinetics of the nitazenes in the 4 major CYPs where they are significantly metabolized is summarized in Table 5.

**TABLE 5:** Metabolic kinetics of butonitazene, isotonitazene and protonitazene in recombinant cytochrome P450 enzymes.

Compound	Matrix	% metabolism	Half-life (min)	Clint (µL/min/100 pmol)
Butonitazene	CYP2D6	99	2	920
	CYP3A4	44	22	64
	CYP2B6	83	10	137
Isotonitazene	CYP2D6	72	9	153
	CYP3A4	53	18	78
	CYP2C8	48	13	108
Protonitazene	CYP2D6	100	1	960
	CYP3A4	54	16	85
	CYP2B6	96	5	290
	CYP2C8	41	44	31
Dextromethorphan	CYP2D6	100	1	1,193
Testosterone	CYP3A4	94	9	154
Bupropion	CYP2B6	58	22	62
Amodiaquine	CYP2C8	82	9	149

### Metabolite identification

The incubation of butonitazene, isotonitazene and protonitazene in human liver micromes and liver S9 fractions was analyzed for metabolite formation and identification. Metabolite profiling samples were analyzed on applied biosystems, ABSciex 4500 triple quadrupole instrument. Several biotransformation pathways were explored including, but not limited to, hydroxylation, dihydroxylation, demethylation, dehydrogenation, desethylation, nitro-reduction, hydrogenation, loss of nitro group, loss of nitrogen, dealkylation, desethylation followed by dealkylation, glucuronide conjugation, sulfate conjugation, methylation, acetylation, glycine conjugation, taurine conjugation, cysteine conjugation, glutathione conjugation and *N*-acetylcysteine conjugation. However, only products of hydroxylation, desethylation, dealkylation, desethylation followed by dealkylation, and desethylation followed by hydroxylation were confirmed in total ion chromatogram (TIC), extracted ion chromatogram (XIC) and similar fragment ions (Table 6).

**TABLE 6:** Metabolite profiling of butonitazene, isotonitazene and protonitazens in human liver microsomes and liver S9 fractions, indicating the presence (√) of putative metabolites.

€Compound	m/z	HLM	HL S9
Butonitazene			
**Parent**	425.255	√	√
Hydroxylation	441.2499	√	√
N-Desethylation	397.2238	√	√
Dealkylation	369.1921	√	√
Desethylation + Dealkylation	341.1908	√	√
Desethylation + Hydroxylation	413.2138	√	√
Isotonitazene			
**Parent**	411.239	√	√
Hydroxylation	427.239	√	√
N-Desethylation	383.208	√	√
Dealkylation	369.171	√	-
N-Desethylation + Dealkylation	341.150	√	√
N-Desethylation + Hydroxylation	399.197	√	√
Depropylated-demethylated	355.176	√	√
N-De-ethylamination and Dealkylation	312.130	√	√
N-desethylation, debutylation	313.130	√	√
Protonitazene			
**Parent**	411.239	√	√
Hydroxylation	427.234	√	√
N-Desethylation	383.208	√	√
Dealkylation	369.180	√	√
N-Desethylated + Dealkylated	341.150	√	√
Demethylated + Dealkylation	355.176	√	√
De-aminopentylated	340.160	√	√
N-Desethylation + Hydroxylation	399.197	√	√

The observed primary metabolites were more clearly visible in human liver microsomes in comparison to the human liver S9 fractions and plasma. This less abundance in the later matrix could be due to further transformations to secondary metabolites or degradation to other undetected forms.

All the identified metabolites are only putative as there are no synthetic standards for confirmation. To probe these metabolites further, the mass spectrometer peak area was used to estimate % relative abundances relative to the area count of the parent compound at 0 min (It should be noted that ionization potential may vary between parent and observed metabolites during mass spectrometry run and analyte area capturing) (Table 7). Although, the analyses of metabolite profiles of butonitazene, isotonitazene and protonitazene were performed in both positive and negative mode of ionization, the metabolites were observed only in the positive mode. Butonitazene, isotonitazene, protonitazene were mainly fragmented to desethylated (up to 50%) and dealkylated (∼3%) parts in *vitro* incubations (Table 7; See also Supplementary Appendix S1–3). Secondary metabolites including glucuronide conjugate formation were not observed despite anticipation of formation in the S9 fractions.

**TABLE 7:** Analysis of the putative metabolites of butonitazene in human liver microsomes and liver S9 fractions.

Ion/Fragment	% relative intensity (sum intensity)	% relative abundance (peak area)	% relative intensity (sum intensity)	% relative abundance (peak area)
	Human liver microsomes		Human liver S9	
Butonitazene				
Hydroxylation	0.4	0.4	0.7	0.7
N-Desethylation	51	50	28	27
Dealkylation	2.5	2.6	1.2	1.4
Desethylation + Dealkylation	16.3	16.3	8.1	8.6
Desethylation + Hydroxylation	1.7	1.9	1.3	1.6
Isotonitazene				
Hydroxylation	0.5	0.5	0.3	0.3
N-Desethylation	27	33	30	25
Dealkylation	0.02	0.02	-	-
N-Desethylation + Dealkylation	0.98	1.20	0.6	0.9
N-Desethylation + Hydroxylation	0.2	0.2	0.1	0.2
Depropylated-demethylated	1.2	1.4	0.9	0.8
N-De-ethylamination and Dealkylation	0.1	0.1	0.1	0.1
N-desethylation, debutylation	0.7	0.8	0.5	0.5
Protonitazene				
Hydroxylation	0.3	0.4	0.5	0.4
N-Desethylation	41	37	31	30
Dealkylation	0.16	0.19	0.6	0.8
N-Desethylated + Dealkylated	0.71	1.05	0.7	0.9
Demethylated + Dealkylated	1.8	1.9	1.0	1.1
De-aminopentylated	0.08	0.09	0.4	0.5
N-Desethylation + Hydroxylation	1.0	1.0	1.0	0.9

## Discussion

Understanding the physiological disposition of new chemical entities is a standard component of early drug discovery. *In vitro* assays provide such insights necessary to estimate the contribution of metabolism to drug elimination, along with information on the enzymes responsible for such metabolism. For several drugs, the pharmacological and toxicological responses are related to metabolism. This relationship can be influenced by multiple factors including pharmacogenomics and concomitantly administered drugs.

There have not been any detailed drug metabolism studies on nitazenes partly because their development was discontinued decades ago. However, their emergence since 2019 in the illicit drug market has drawn interests in their pharmacology and disposition. In a previous study, products of *N*- and *O*-dealkylation products of isotonitazene were detected in forensic samples (Krotulski et al., 2020). More recently, Taoussi et al. (2024) reported the identification of multiple metabolites isotonitazene, metonitazene, etodesnitazene, and metodesnitazene following incubation with human hepatocytes.

In the current study, the three nitazenes studied (butonitazene, isotonitazene and protonitazene) were rapidly metabolized in human liver microsomes and the human liver S9 fractions. For example, with an *in vitro* intrinsic clearance of 309 μL/min/mg protein, and a half-life of only 4 min, butonitazene had more than double the *in vitro* elimination rate of verapamil (*in vitro* intrinsic clearance of 150 μL/min/mg protein, and *in vitro* half-life of 9 min). Similarly, the elimination of the nitazenes in the liver S9 fraction was very rapid with a rate as high as 6 times that of testosterone, the standard positive control. Compared to testosterone at 40 min, the 3 nitazenes had half-life values to 10 min or less. These values from microsomal and S9-fraction metabolism suggest that nitazenes will be subjected to liver-dependent elimination.

The observed rapid *in vitro* depletion of the nitazenes can provide some initial insights into the roles of pharmacokinetics on their action and analysis. Drugs that are rapidly metabolized may have short duration of action unless the metabolites are active. Exceptions occur for drugs whose transient interaction with receptors induces biochemical changes that result in longer-lasting effect. Thus, our current understanding of the receptor pharmacology of nitazenes may be incomplete without an adequate evaluation of the activities, if any, of the metabolites. The rapid metabolism also has implications for timely detection and quantification in human matrices. Due to their potent nature, it is expected that nitazenes are consumed in microgram quantities. This, in addition to rapid clearance will require a timely post-ingestion sampling and highly sensitive analytical methodology for detection and quantitation. In a compilation of acute intoxications and fatalities associated with nitazenes in humans, detected levels were in single unit nanogram per milliliter range. While this may reflect the potency of nitazenes with regards to the amount consumed, it may also reflect its rapid elimination (Montanari et al., 2022; Walton et al., 2023). In a previous study, products of *N*- and *O*-dealkylation products of isotonitazene were detected in forensic samples (Krotulski et al., 2020).

The rapid metabolism of nitazenes in hepatic matrices may also have implications for the detection of the unchanged compounds in the urine and other body matrices. This is important because most forensic analysis of drugs of abuse rely heavily on urinary analysis. While the detection of metabolites in the urine can be useful in determining the identities of the abused substance, the diversity, and structural similarities among the nitazenes may make this challenging.

The metabolic phenotyping of nitazenes showed interesting results too. CYP2D6, CYP2B6 and CYP2C8 appeared to be the primary CYPs responsible for the metabolism of nitazenes. Previously known and well-studied opioids include those that belong to the phenanthrene, phenylpiperidine or diphenylheptane chemical classes. To varying degrees, all known opioids are metabolized by liver enzymes (Smith, 2009). Most phenanthrene opioids (codeine, hydrocodone, oxycodone) are primarily metabolized by CYP2D6 with contributions from CYP3A4 (codeine and oxycodone). The phenylpiperidine opioids (fentalogues) and diphenylheptanes are primarily metabolized by CYP3A4. Thus, the chemical diversification of opioids has implications for metabolic enzyme substrate affinity. CYP2D6 is one of the most polymorphic metabolic enzymes. Its metabolic phenotypes, with implications for pharmacotherapy and toxicity, have been well established (Mikus and Weiss (2005). For example, as a prodrug whose activation is CYP2D6-dependent, codeine is not an analgesic of choice in individuals identified with CYP2D6 poor or ultra-rapid phenotypes. While the inability to convert codeine to the active morphine (poor metabolizers) would result in poor pain control, rapid activation can lead to respiratory depression and other toxic manifestations due to excessive morphine effect. CYP-dependent metabolism of nitazenes may have pharmacogenomic implication for susceptibility to intoxication and/or addiction. CYP2B6 and CYP2C8 are polymorphic and response to nitazenes in individuals may be genetically influenced. Over 38 known variant alleles of *CYP2B6* genes have been reported resulting in activity phenotypes of slow, normal, and ultrarapid metabolizers of such important drug substrates as efavirenz and sertraline (Desta et al., 2019; Bousman et al., 2023). Similar genetic variations in the expressions of *CYP2C8* gene have shown distinct metabolic phenotypes of drugs like diclofenac and ibuprofen (Theken et al., 2020). Additionally, several important drugs are metabolized by CYP2D6, CYP2B6 and CYP2C8. There is, therefore, a strong potential for drug-drug interaction in cases of concomitant drug administrations.

This study identified several putative metabolic products. While the suggested metabolites are not confirmed, products of ring hydroxylation and dealkylation are expected based on the chemical structure of nitazenes. These putative metabolites can be subjects of further evaluation to confirm identity and any pharmacological activity. It should be noted that some of these metabolites have earlier been detected in forensic samples (Taoussi et al., 2024).

## Conclusion

Nitazenes are rapidly biotransformed through phase 1 (CYP) enzymes in HLM, and HL S9 fractions. The primay CYP enzymes responsible for the metabolism of nitazenes are CYP2D6, CYP2B6 and CYP 2C8. Several putative products of hydroxylation and dealkylation were identified. The rapid metabolism of nitazenes may have implications in the detections and quantitation of the unchanged drugs in human matrices following intoxication or in forensic analysis. For a more holistic understanding of the pharmacology of nitazenes, it may be necessary to further evaluate the potential activity of their metabolites.

## Data Availability

The original contributions presented in the study are included in the article/Supplementary Material, further inquiries can be directed to the corresponding author.
