# Supplementary material for: Metabolic characterization of the new benzimidazole synthetic opioids - nitazenes
Source: Front Pharmacol. 2024 Jul 18;15:1434573. doi: 10.3389/fphar.2024.1434573 (PMC11291330; doi:10.3389/fphar.2024.1434573)
Supplement: Supplementary file 1 [file Table1.DOCX]

Supplementary Materials

**Appendix A. Fragmentation pattern of butonitazene in uman liver microsome (HLM) and human liver S9 (HS9) fraction**

| **Metabolite** | **m/z** | **Retention time (min)** | **Fragmentation** |
| --- | --- | --- | --- |
| Human Liver Microsomes | | | |
| Parent | 425.255 | 5.534 | 114.0910, 130.1586, 141.1131, 200.2378, 201.2404, 213.1314, 425.2548, 426.2585, 593.3061, 610.3330, 615.2885 |
| Hydroxylation | 441.2499 | 4.169 | 60.0439, 88.0751, 114.0908, 130.1586, 141.1128, 158.1899, 200.2371, 215.1251, 251.1854, 275.2578, 309.2267, 441.2494 |
| N-Desethylation | 397.2238 | 5.324 | 200.2371, 201.2399, 341.1608, 397.2218, 398.2218, |
| Dealkylation | 369.1921 | 5.081 | 60.0439, 114.0909, 130.1587, 141.1132, 200.2375, 251.1853, 275.2588, 313.1305, 369.1926, 368.2647, 496.3399, 535.2647, 536.2677, 537.2706, 552.2912, 553. 2942, 557.2465, 659. 2706 |
| Desethylation + Dealkylation | 341.1908 | 5.328 | 200.2369, 201.2399, 341.1908, 397.2218, 659.2687 |
| Desethylation + Hydroxylation | 413.2138 | 3.985 | 54.0082, 60.0440, 88.0752, 114.0910, 130.1588, 141.1134, 193.1432, 200.2377, 261.2428, 251.1856, 275.2585, 310.2290, 309.2276, 319.2847, 354.2850, 414.2214, 413. 2184, 435.1766, 452.2030 |
| Human Liver S9 | | | |
| Parent | 425.255 | 5.564 | 114.0910, 130.1586, 141.1131, 200.2378, 201.2404, 213.1314, 425.2548, 426.2585, 593.3061, 610.3330, 615.2885 |
| Hydroxylation | 441.2499 | 4.189 | 54.0083, 60.0441, 100.1114, 114.0911, 130.1589, 141.1133, 193.1437, 200.2379, 215.1260, 223.0643, 251.1860, 275.2587, 309.2276, 435.1770, 441.2501, 442.2524 |
| N-Desethylation | 397.2238 | 5.352 | 200.2380, 201.2410, 397.2239, 398.1051, 491.2351 |
| Dealkylation | 369.1921 | 5.104 | 60.0439, 114.0909, 130.1587, 141.1132, 200.2375, 251.1853, 275.2588, 313.1305, 369.1926, 368.2647, 496.3399, 535.2647, 536.2677, 537.2706, 552.2912, 553. 2942, 557.2465, 659. 2706 |
| Desethylation + Dealkylation | 341.1608 | 5.349 | 200.2380, 201.2411, 341.1618, 399.1201, 498.2310, 659.2725 |
| Desethylation + Hydroxylation | 413.2138 | 3.997 | 54.0082, 60.0440, 88.0752, 114.0910, 130.1588, 141.1134, 193.1432, 200.2377, 261.2428, 251.1856, 275.2585, 310.2290, 309.2276, 319.2847, 354.2850, 414.2214, 413. 2184, 435.1766, 452.2030 |

**Appendix B. Fragmentation pattern of isotonitazene in HLM, and HS9**

| **Metabolite** | **m/z** | **Retention time (min)** | **Fragmentation** |
| --- | --- | --- | --- |
| Human Liver Microsomes | | | |
| Parent | 411.239 | 4.941 | 114.0907, 185.1002, 206.1239, 411.2406, 412.2436, 821.4731 |
| Hydroxylation | 427.234 | 4.918 | 114.0908, 185.1003, 206.1241, 206.6258, 411.2405, 412.2436, 427.2337, 535.2658, 536.2685, 821.4730 |
| N-Desethylation | 383.208 | 4.714 | 114.0908, 130.1586, 200.2378, 341.1615, 383.2090, 384.2122, 477.2244 |
| Dealkylation | 368.170 | 3.751 | 60.0439, 88.0752, 114.0911, 130.1589, 141.1134, 155.1287, 193.1437, 200.2379, 215.1260, 251.1862, 261.2432, 273.1683, 275.2590, 297.2412, 319.2898, 355.166, 384.2967, 477.2248, 496.3411 |
| N-Desethylation Dealkylation | 341.150 | 3.172 | 54.0082, 60.0440, 88.0751, 114.0909, 130.1587, 141.1131, 193.1434, 200.2374, 215.1256, 251.1853, 273.1672, 341.1614, 383.2080, 384.2110, 477.2229, 494.2495 |
| N-Desethylation + Hydroxylation | 399.197 | 3.714 | 60.0437, 88.0750, 114.0910, 130.1588, 141.1131, 193.1437, 200.2377, 215.1260, 233.0639, 251.1861, 275.2590, 273.1680, 319.2853, 333.3010, 399.1663 |
| Depropylated-demethylated | 355.176 | 4.463 | 60.0439, 88.0752, 114.0911, 130.1589, 141.1134, 155.1287, 193.1437, 200.2379, 215.1260, 251.1862, 261.2432, 273.1683, 275.2590, 297.2412, 319.2898, 355.166, 384.2967, 477.2248, 496.3411 |
| N-De-ethylamination and Dealkylation | 312.130 | 4.720 | 60.0436, 88.0748, 114.0907, 130.1587, 171.0840, 200.2379, 215.1260, 251.1861, 273.1679, 275.2588, 312.1328, 319.2849, 341.1615, 342.1641, 383.2089, 384.2123, 477.2244, 494.2511 |
| N-desethylation, debutylation | 313.130 | 4.470 | 54.0083, 60.441, 88.0753, 114.0911, 130.1588, 141.1133, 155.1287, 193.1430, 200.2374, 215.1256, 251.1852, 261.2427, 273.1675, 275.2581, 319.2848, 367.2691, 384.2960, 389.2509, 477.2235 |
| Human Liver S9 | | | |
| Parent | 411.239 | 4.954 | 60.0440, 114.0904, 130.1583, 141.1127, 185.100, 200.2372, 206.1231, 225.1022, 411.2389, 412.2424, 535.2642, 659.2695 |
| Hydroxylation | 427.2375 | 4.189 | 60.0440, 114.0905, 130.1584, 141.1128, 200.2374, 215.1249, 251.1853, 275.2580, 375.2160, 411.2390, 412.2426, 535.2644, 536.2674, 557.2459, 659.2698 |
| N-Desethylation | 383.2079 | 4.738 | 54.0082, 60.0440, 88.0750, 114.0909, 130.1587, 141.1131, 193.1433, 200.2374, 215.1256, 251.1853, 273.1671, 275.2582, 319.2843, 341.1612, 383.2079, 477.2227, 494.2493 |
| N-Desethylation + Dealkylation | 341.150 | 3.172 | 54.0082, 60.0440, 88.0751, 114.0909, 130.1587, 141.1131, 193.1434, 200.2374, 215.1256, 251.1853, 273.1672, 341.1612, 383.2080, 384.2110, 477.2229, 478.2265, 494.2495 |
| N-Desethylation + Hydroxylation | 399.190 | 3.741 | 60.0437, 88.0750, 114.0910, 130.1588, 141.1131, 193.1437, 200.2377, 215.1260, 223.0639, 251.1861, 275.2590, 273.1680, 319.2853, 333.3010 |
| Depropylated-Demethylated | 355.1769 | 4.481 | 54.0082, 60.0441, 88.0752, 114.0910, 130.1587, 141.1132, 155.1286, 193.1432, 200.2374, 215.1256, 217.1058, 251.1854, 261.2423, 273.1672, 275.2583, 297.2406, 319.2847, 355.1770, 367.2689, 384.2959, 389.2507, 477.2226 |
| N-De-ethylamination and Dealkylation | 312.130 | 4.720 | 60.0436, 88.0748, 114.0907, 130.1587, 171.0840, 200.2379, 215.1260, 251.1861, 273.1679, 275.2588, 312.1328, 319.2849, 341.1615, 342.1641, 383.2089, 384.2123, 477.2244, 494.2511 |
| N-desethylation, debutylation | 313.120 | 4.471 | 54.0083, 60.441, 88.0753, 114.0911, 130.1588, 141.1133, 155.1287, 193.1430, 200.2374, 215.1256, 251.1852, 261.2427, 273.1675, 275.2581, 319.2848, 367.2691, 384.2960, 389.2509, 477.2235 |

**Appendix C. Fragmentation pattern of protonitazene in HLM, and HS9**

| **Metabolite** | **m/z** | **Retention time (min)** | **Fragmentation** |
| --- | --- | --- | --- |
| Human Liver Microsomes | | | |
| Parent | 411.239 | 5.129 | 200.2372, 201.2401, 206.1230, 411.2387, 412.2616 |
| Hydroxylation | 427.234 | 3.898 | 54.0081, 60.0439, 88.0750, 114.0909, 130.1585, 141.1130, 171.1487, 193.1430, 200.2371, 215.1253, 251.1853, 251.2421, 273.1669, 275.2579, 309.2268, 331.2086, 427.2346, 435. 1755 |
| N-Desethylation | 383.208 | 4.909 | 114.0908, 383.2075, 535.2639 |
| Dealkylated | 369.180 | 3.431 | 60.0439, 88.0751, 114.0910, 130.1589, 141.1133, 177.0546, 200.2375, 215.1258, 251.1858, 252.1874, 273.1677, 275.2587, 317.1501, 318.1534, 319.2847, 350.3262, 369.1989, 373.5537, 405.0091, 435.1760, 436.1796, 529.4001, 564.3595, 598.0686, 746.0980 |
| N-Desethylated + Dealkylated | 341.150 | 3.188 | 60.0439, 88.0751, 114.0910, 130.1589, 141.1131, 149.0230, 193.1434, 215.1258, 251.1858, 273.1678, 275.2585, 317.1500, 341.1613, 373.5532 |
| Demethylated + Dealkylation | 355.176 | 4.663 | 54.0083, 60.0440, 88.0752, 114.0908, 130.1585, 141.1130, 193.1428, 200.2373, 215.1253, 261.2424, 251.1852, 273.1671, 275.2581, 319.2842, 355.1766, 383.2076, 477.2223, 478.2251, 494.2483, 535.2637, 659.2701 |
| De-aminopentylated | 340.160 | 4.755 | 60.0440, 88.0748, 114.0908, 130.1586, 141.1131, 192.1079, 200.2372, 225.1025, 251.1857, 273.1673, 341.1611, 383.2080, 384.2110, 477.2231, 478.2263, 496.3393, 535.2645, 659.2696 |
| Desethylation + Hydroxylation | 399.197 | 3.668 | 60.0439, 88.0751, 114.0911, 130.1589, 141.1132, 193.1434, 200.2374, 215.1259, 251.1858, 275.2587, 317.1504, 375.2621, 399.2028, 435.1764 |
| Human Liver S9 | | | |
| Parent | 411.239 | 5.133 | 200.2375, 201.2409, 411.2396, 535.2655 |
| Hydroxylation | 427.234 | 3.896 | 60.0440, 88.0753, 114.0910, 130.1587, 141.1132, 193.1434, 200.2374, 223.0639, 251.1856, 275.2585, 309.2279, 331.2090, 375.2625, 375.7678, 427.2339, 435.1766, 452.2044, 749.5170, 750.5210 |
| N-Desethylation | 383.208 | 4.920 | 54.0081, 60.0441, 88.0751, 114.0910, 130.1588, 141.1131, 155.1287, 200.2375, 225.1027, 251.1857, 273.1674, 275.2582, 319.2849, 383.2076, 496.3390, 535.2653, 557.2647, 659.2713, 660.2746 |
| Dealkylated | 369.180 | 3.431 | 60.0439, 59.0484, 88.0751, 114.0910, 130.1589, 141.1133, 177.0546, 200.2375, 215.1258, 251.1858, 252.1874, 273.1677, 275.2587, 317.1501, 318.1534, 319.2847, 350.3262, 369.1931, 373.5537, 405.0091, 435.1760, 436.1796, 529.4001, 564.3595, 598.0686, 746.0980 |
| N-Desethylated + Dealkylated | 341.150 | 3.188 | 59.0487, 60.0440, 88.0752, 114.0912, 130.1590, 141.1133, 177.0549, 200.2374, 215.1257, 251.1862, 275.2584, 297.2411, 319.2848, 341.1602, 350.3263, 373.5532, 405.0101, 435.1774, 520.3328, 564.3328, 564.3577, 581.0410, 598.0676, 657.6872, 746.0977 |
| Demethylated + Dealkylation | 355.176 | 4.661 | 54.0082, 60.0440, 88.0751, 114.0910, 130.1589, 141.1133, 174.1027, 193.1434, 200.2377, 215.1259, 223.0636, 251.1857, 275.2586, 319.2848, 355.1762, 367.2898, 477.2235, 478.2271, 494.2501 |
| De-aminopentylated | 340.160 | 4.744 | 54.0083, 60.0440, 88.0752, 114.0910, 130.1589, 141.1133, 155.1288, 174.1028, 200.2377, 225.1029, 251.1859, 273.1679, 275.2587, 319.2847, 340.1768, 383.2089, 477.2235, 478.2272, 494.2503 |
| Desethylation + Hydroxylation | 399.198 | 3.664 | 60.0439, 88.0751, 114.0911, 130.1589, 141.1132, 193.1434, 200.2374, 215.1259, 251.1858, 275.2587, 317.1504, 375.2621, 399.2028, 435.1764 |
